# Nine primary malignant neoplasms-involving the esophagus, stomach, colon, rectum, prostate, and external ear canal-without microsatellite instability: a case report

**DOI:** 10.1186/s12885-017-3973-2

**Published:** 2018-01-04

**Authors:** Keiichi Arakawa, Keisuke Hata, Yoko Yamamoto, Takeshi Nishikawa, Toshiaki Tanaka, Tomomichi Kiyomatsu, Kazushige Kawai, Hiroaki Nozawa, Masafumi Yoshida, Hiroshi Fukuhara, Mitsuhiro Fujishiro, Teppei Morikawa, Tatsuya Yamasoba, Kazuhiko Koike, Masashi Fukayama, Toshiaki Watanabe

**Affiliations:** 1 Department of Surgical Oncology, the University of Tokyo, Tokyo, Japan; 2 Department of Otolaryngology, the University of Tokyo, Tokyo, Japan; 3 Department of Urology, the University of Tokyo, Tokyo, Japan; 4 Department of Gastroenterology, the University of Tokyo, Tokyo, Japan; 5 Department of Pathology, the University of Tokyo, Tokyo, Japan; 6 Department of Surgical Oncology, University of Tokyo Hospital, 7-3-1 Hongo, Bunkyo-ku, Tokyo, 113-0033 Japan

**Keywords:** Multiple primary neoplasms, Microsatellite instability, Colorectal neoplasms

## Abstract

**Background:**

Although cases of multiple primary malignant neoplasms are increasing, reports of more than three or four primary metachronous malignant neoplasms are extremely rare. Moreover, very few publications have provided a genetic mutational analysis or have evaluated risk factors associated with such neoplasms. We present an extremely rare case of nine primary malignant lesions in a man who was successfully treated. We also report on microsatellite stability status, analyze risk factors, and discuss the relevant literature.

**Case presentation:**

Between 67 and 73 years of age, a male patient developed nine primary metachronous malignant lesions: Three were located in the esophagus, two in the stomach, two in the colorectum, one in the prostate gland, and one in the external ear canal. The patient’s clinical history included hypertension, atrial fibrillation, an acoustic schwannoma, and heavy smoking. The lesions were diagnosed during regular screening over a six-year period. He was successfully treated with surgery (both open surgical and endoscopic resection of lesions) and adjuvant chemotherapy. Immunohistochemistry and mutational analysis showed that the lesions were microsatellite stable, and the *KRAS*, *BRAF*, p53, and nuclear β-catenin status was not uniform among the lesions.

**Conclusions:**

Given that the presence of more than three or four neoplasms is extremely rare, the present case of nine primary malignancies with no associated microsatellite instability and no apparent predisposing hereditary conditions, is extraordinary. Our case study shows that it is possible for up to nine sporadic neoplasms to occur, and efficient disease management requires diligent screening and early detection.

**Electronic supplementary material:**

The online version of this article (10.1186/s12885-017-3973-2) contains supplementary material, which is available to authorized users.

## Background

The incidence of multiple primary malignant neoplasms (MPMNs) is rising, partly due to medical advances in diagnostic and therapeutic strategies that prolong survival, allowing other primary cancers to develop [1]. It is estimated that 0.7–11.7% of patients with malignancies develop MPMNs [1]. Double malignant neoplasms are reported to occur in 2.2% of all cases of colorectal cancer, while triple cancers occur in 0.5% of MPMN cases. Four or more neoplasms occur in <0.1% of MPMN cases [2]. There are only two case reports of patients with eight primary malignant neoplasms [3, 4]. To our knowledge, no cases with nine or more primary malignant neoplasms have been reported in the literature. We present an extremely rare case of nine primary metachronous and synchronous malignant neoplasms in one patient, involving the esophagus, stomach, colon, rectum, prostate, and external ear canal.

## Case presentation

Between the ages of 67 and 73 years, a male patient developed nine MPMNs: Three lesions were located in the esophagus, two in the stomach, two in the colorectum, one in the prostate gland, and one in the external ear canal (Table 1). An esophageal lesion was first detected via a screening gastroscopy; the other eight lesions were found during screening examinations over the subsequent six years. The three esophageal and two gastric lesions were successfully removed by endoscopic submucosal dissection, while the two colorectal lesions and the lesion in the external ear canal were surgically resected. Total colonoscopy, performed before the patient underwent rectal surgery, did not detect any tumors in sigmoid colon. Adjuvant chemotherapy (tegafur/uracil) was administered after resection of the sigmoid colon tumor. Currently, two years post-surgery, the patient is relapse-free and attends outpatient follow-up.

**Table 1:** Clinicopathologic characteristics of the patient

Age	Organ	Treatment	Histology	TNM stage^a^
67	Esophagus 1	ESD	Squamous cell carcinoma	TisN0M0
67	Esophagus 2	ESD	Squamous cell carcinoma	TisN0M0
67	Stomach 1	ESD	Well differentiated adenocarcinoma	TisN0M0
69	Ear canal	Surgical resection	Squamous cell carcinoma	T2N0M0
72	Esophagus 3	ESD	Squamous cell carcinoma	T1N0M0
72	Rectum	Surgical resection	Moderately differentiated adenocarcinoma	T2N0M0
73	Stomach 2	ESD	Well differentiated adenocarcinoma	T1N0M0
73	Prostate	Observation	Well differentiated adenocarcinoma	T1N0M0
73	Colon	Surgical resection	Well differentiated adenocarcinoma	T4N0M0

ESD: endoscopic submucosal dissection

^a^Staging was performed according to the Union for International Cancer Control TNM Classification of Malignant Tumors (7th edition)

The patient’s clinical history included hypertension, atrial fibrillation, and, at 58 years of age, vagus nerve resection for an acoustic schwannoma. He was an office worker and had smoked heavily (one pack per day) for 30 years. He also experienced facial flushing after consuming small volumes of alcohol; he consumed little alcohol. His family history revealed that his younger sister had had esophageal cancer and his mother had had ovarian cancer. He tested positive for *Helicobacter pylori* (*H. pylori)*, although this infection had been successfully eradicated when he was 67 years old.

Formalin-fixed paraffin-embedded (FFPE) tissue from the nine lesions was subjected to immunohistochemistry (IHC) testing for p53 and nuclear β-catenin expression. The IHC analysis was performed using an anti-p53 antibody (Clone DO-7; Dako, Glostrup, Denmark) and an anti-β-catenin antibody (BD Transduction Laboratory, San Diego, CA, USA). In addition, somatic mutational analysis (for *KRAS*, *BRAF*, and microsatellite instability [MSI]) was performed [5]. Using the QIAamp DNA FFPE tissue kit (Qiagen, Valencia, USA), genomic DNA was extracted from each FFPE tissue specimen. MSI was analyzed by polymerase chain reaction at five microsatellite loci: BAT25, BAT26, D2S123, D5S346, and D17S250, as previously described [6]. Direct sequencing of the extracted DNA was performed to evaluate mutations in *KRAS G12*, *G13*, and *BRAF V600* [6]. In addition, direct sequencing of extracted DNA from normal mucosa was performed to evaluate single nucleotide polymorphisms in *ALDH2* and *ADH1B* [7, 8].

**Fig. 1:**
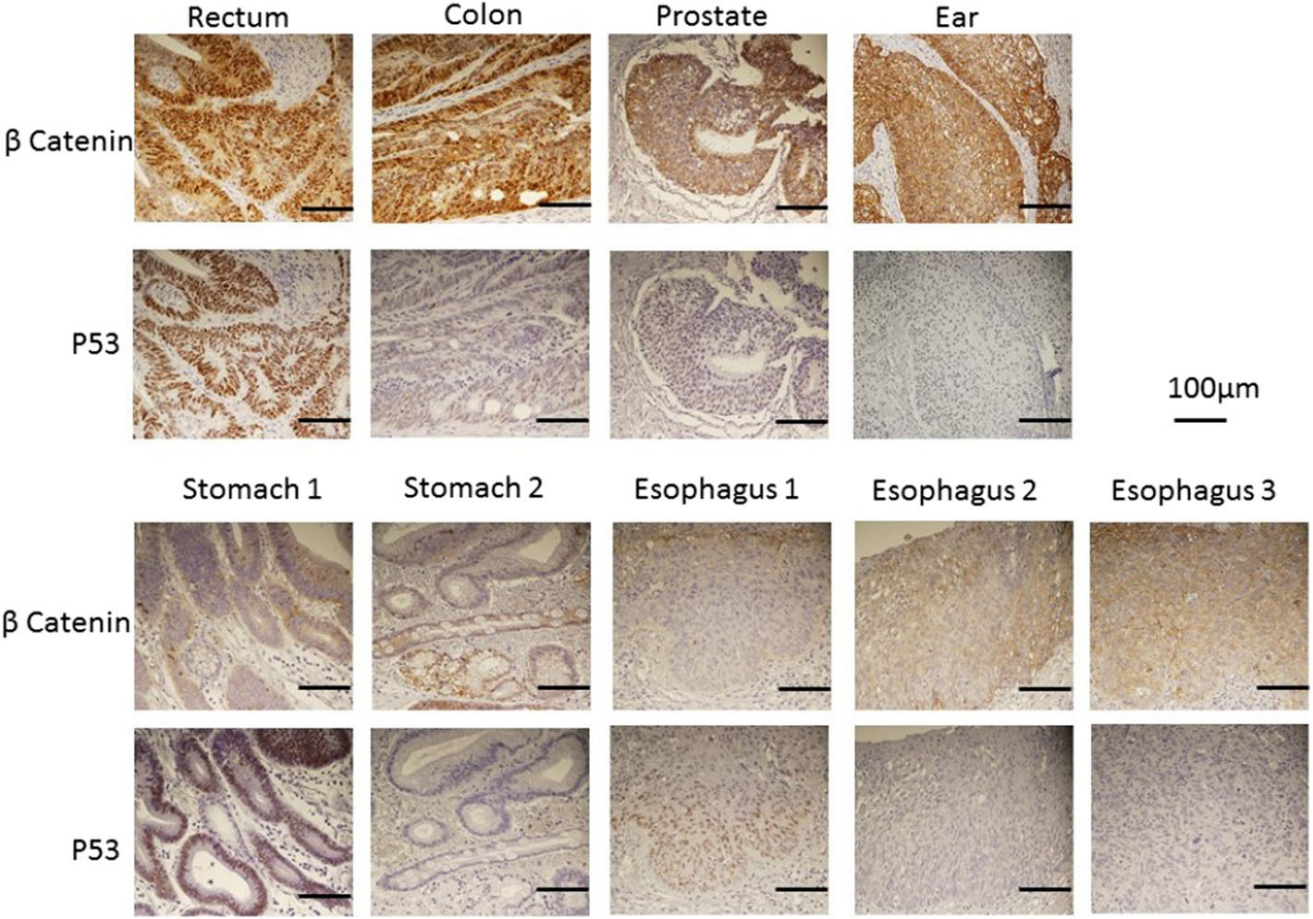
Representative images of the immunohistochemical (IHC) analysis of lesions isolated from a man with an extremely rare case of nine primary malignant neoplasms, using anti-β-catenin and anti-p53 antibodies. The colonic and rectal lesions were nuclear β-catenin positive. The colonic and initial gastric and esophageal lesions were p53 positive

The results of the IHC, somatic mutational, and MSI analyses are listed in Table 2. Representative IHC images are presented in Fig. 1. The first examined esophageal, gastric, and rectal lesions were p53 positive, while the colonic and rectal lesions were nuclear β-catenin positive; all remaining lesions were p53 and nuclear β-catenin negative. All lesions were microsatellite stable (MSS) (Additional file 1: Figure S1).

**Table 2:** Histologic types and genetic characteristics of the cancers

Organ	Histology	Immunohistochemistry		Mutation		MSI
		p53	Nuclear β-catenin	*KRAS*	*BRAF*	
Esophagus 1	SCC	+	–	Wt	Wt	MSS
Esophagus 2	SCC	–	–	Wt	Wt	MSS
Stomach 1	AC	+	–	Wt	Wt	MSS
Ear canal	SCC	–	–	Wt	Wt	MSS
Esophagus 3	SCC	–	–	Wt	Wt	MSS
Rectum	AC	+	+	G12C	Wt	MSS
Stomach 2	AC	–	–	G12C	Wt	MSS
Prostate	AC	–	–	Wt	Wt	MSS
Colon	AC	–	+	G13 V	Wt	MSS

The histologic, immunohistochemical (p53, β-catenin), and mutational analysis (*KRAS*, *BRAF*, and microsatellite status) characteristics of surgically and endoscopically removed tumor tissues from a man diagnosed with nine metachronous primary malignant lesions

SCC, squamous cell carcinoma; AC, adenocarcinoma; Wt, wild type; MSI, microsatellite instability; MSS, microsatellite stable

The study protocol was approved by the Ethical Review Board committee of our hospital and the research was conducted according to the Declaration of Helsinki. The patient provided written informed consent for publication.

## Discussion and conclusions

To the best of our knowledge, the highest number of malignant lesions ever reported in one patient is eight [3, 4]; this is the first case report of nine primary malignant lesions occurring in a single patient. Genetic abnormalities and the status of mutations associated with increase in cancer risk have been evaluated in a few cases of MPMN. MSI indicates increased hypermutability of the genome and is reliably associated with various carcinomas, with colon cancer being the most closely linked; MSI could be a common denominator in the development of multiple neoplasms. The literature contains two case reports of five and six primary malignancies in which the patients’ MSI status was studied. The patient with five neoplasms—adenocarcinoma and neuroendocrine carcinoma of the stomach, jejunum, ascending colon, transverse colon, and rectum—had MSI [9]. In the patient with six neoplasms—carcinomas of the rectum, urinary bladder, stomach, descending colon, liver, and lung—replication errors and instability in seven microsatellite markers were found; MSI was present [10]. In contrast, when we investigated the MSI status of each tumor, we found that all the lesions were MSS, ruling out errors in DNA mismatch repair as a possible causative factor in this case. In addition to microsatellite stability status, tumor protein p53 and nuclear β-catenin expression status are also closely linked to carcinogenesis. In our case study, IHC demonstrated that p53 status was positive in the esophageal, gastric, and rectal tumors at first treatment but was negative in the esophageal cancers at the time of second and third treatments, in the gastric cancer at second the treatment, and in the cancers of the colon, prostate, and external ear canal. In contrast, nuclear β-catenin was positive in the colon and rectal cancers, indicating a possible “alternate pathway of carcinogenesis” [11].

The extreme rarity of the occurrence of nine neoplasms led us to hypothesize that the patient may have a genetic susceptibility to MPMN, such as a hereditary gastrointestinal syndrome, including Lynch Syndrome. This patient had seven carcinomas in the gastrointestinal tract—with lesions in the esophagus, stomach, and colorectum—and a tumor in the prostate gland and external ear canal. The major relevant hereditary gastrointestinal cancer syndromes are familial adenomatous polyposis (FAP), Lynch syndrome, hereditary diffuse gastric cancer, and Li-Fraumeni syndrome [12].

Patients with FAP exhibit colorectal polyposis and usually harbor adenomatous polyposis coli (*APC*) gene mutations. The deletion at codon 1309 in the germline of the *APC* gene in patients with FAP has been previously reported, especially in profuse-type FAP [13]. The APC protein is a tumor suppressor that interacts with and controls the intracellular concentration of nuclear β-catenin protein by targeting it for degradation. In the present case, colorectal lesions were nuclear β-catenin positive, implying a possible mutation in the *APC* gene. The patient did not have the typical FAP phenotype of polyposis or a nuclear β-catenin positive status; specifically, there was inconsistency in nuclear β-catenin status among the nine lesions. Thus, the diagnosis of FAP was unlikely.

Lynch syndrome is the most common type of hereditary colorectal cancer, with a conservative estimated incidence of 5–10% of all colorectal cancers [14]. In the present case, the patient had metachronous colorectal cancers and a family history of ovarian cancer; both are associated with Lynch syndrome according to the Revised Bethesda guidelines. However, it should be mentioned that this does not conform to the Revised Amsterdam II criteria. Analysis of microsatellite status demonstrated that all nine malignant lesions were MSS, ruling out Lynch syndrome.

Li-Fraumeni syndrome and hereditary breast and ovarian syndrome were unlikely in the present case, since *p53* mutation status was negative, as detected by IHC. However, we could not conclusively rule out a hereditary cancer syndrome as we did not perform germline exome sequencing. Moreover, it is possible that the patient presented with an as yet unknown hereditary cancer syndrome. We did not detect any *BRAF* mutations or MSI, which might indicate a non-hypermutated tumor type [15]. The determination of *KRAS* and *BRAF* genotypes are vital in terms of individualizing molecular targeted therapy [16]. Two colorectal cancers in the present case harbored *KRAS* mutations in conjunction with nuclear β-catenin positivity, indicating an “alternate pathway of carcinogenesis” [11]. We performed total colonoscopy prior to rectal surgery; no lesion was detected in the sigmoid colon at that time. The fact that the new tumor was detected in the sigmoid colon within one year after rectal surgery indicates the possibility of accelerated carcinogenesis or that an interval cancer was overlooked by the previous colonoscopy. However, the patient has not had any subsequent colorectal cancers (since his most recent operation) and does not have any hereditary gastrointestinal cancer syndromes. Therefore, there is little evidence supporting accelerated carcinogenesis in this patient. When combined, the results from microsatellite, *KRAS*, *BRAF*, p53, and nuclear β-catenin studies indicated that the tumors of the stomach, esophagus, prostate gland, and external ear canal might have been sporadic in nature, rather than hereditary.

Longer lifespans, medical advances, and lifestyle habits may result in the observed increasing rate of MPMN. Lifestyle habits associated with carcinogenesis, such as tobacco and alcohol use, may cause multiple independent malignant foci to develop in the epithelium, especially in the head and neck regions and in the esophagus. In the present case, the patient was a heavy smoker and a so called “flusher”. He consumed little alcohol. Patients with *ALDH2* gene mutations are known to have a “flusher” phenotype and are reported to be prone to developing esophageal cancer [17]. Moreover, several studies have reported that genetic polymorphisms of *ALDH2* and *ADH1B* are associated with gastric cancer and colorectal cancer [7, 8]. The patient’s single nucleotide polymorphisms in *ALDH2* and *ADH1* may have accounted for carcinogenesis in six of his cancers, including the esophageal, gastric and colorectal cancers. The organism *H. pylori* is a recognized type I carcinogen for gastric cancer, and the patient in our study had a confirmed *H. pylori* positive status; however, his *H. pylori* infection was successfully eradicated at the age of 67 years.

In conclusion, while the presence of more than three or four neoplasms is extremely rare, the present case of nine primary malignancies with no associated MSI is extraordinary. This case study shows that with diligent screening and follow-up, successful disease management of such cases is possible.

## Additional file

Additional file 1: Figure S1. Tissue from nine lesions isolated from a male patient with MPMNs was analyzed for microsatellite status, and results for markers BAT25 and BAT26 are shown. The microsatellite status was microsatellite stable in all nine lesions in this case. (JPEG 71 kb)

## Abbreviations

ADH1B: alcohol dehydrogenase
ALDH2: acetaldehyde dehydrogenase
APC: adenomatous polyposis coli
DNA: deoxyribose nucleic acid
FAP: familial adenomatous polyposis
FFPE: formalin fixed paraffin embedded
*H. pylori*: *Helicobacter pylori*
IHC: immunohistochemistry
MPMN: multiple primary malignant neoplasms
MSI: microsatellite instability
MSS: microsatellite stable
