# Supplementary figures and images for: Nine primary malignant neoplasms-involving the esophagus, stomach, colon, rectum, prostate, and external ear canal-without microsatellite instability: a case report
Source: BMC Cancer. 2018 Jan 4;18:24. doi: 10.1186/s12885-017-3973-2 (PMC5753511; doi:10.1186/s12885-017-3973-2)

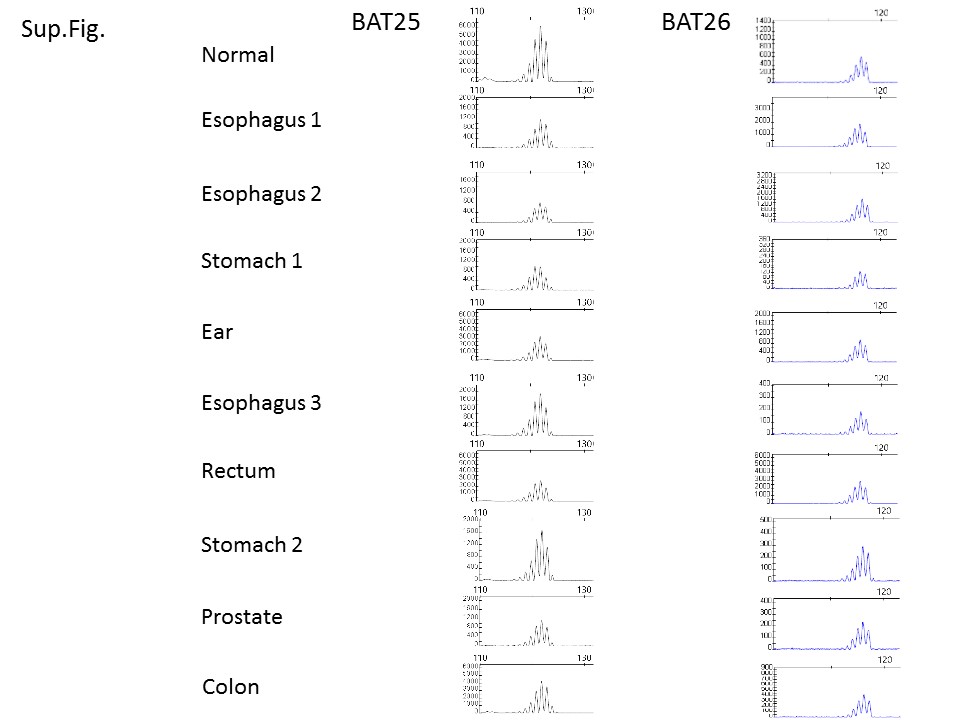

Supplement: Additional file 1: Figure S1. — Tissue from nine lesions isolated from a male patient with MPMNs was analyzed for microsatellite status, and results for markers BAT25 and BAT26 are shown. The microsatellite status was microsatellite stable in all nine lesions in this case. (JPEG 71 kb) [file 12885_2017_3973_MOESM1_ESM.jpg]
